# Supplementary material for: Finding the right tool: a comprehensive evaluation of microglial inducible cre mouse models
Source: bioRxiv. 2023 Apr 17:2023.04.17.536878. Preprint. [Version 1] doi: 10.1101/2023.04.17.536878 (PMC10153116; doi:10.1101/2023.04.17.536878)
Supplement: Supplement 1 [file NIHPP2023.04.17.536878v1-supplement-1.pdf]

bioRxiv preprint doi: https://doi.org/10.1101/2023.04.17.536878; this version posted April 17, 2023. The copyright holder for this preprint (which was not certified by peer review) is the author/funder, who has granted bioRxiv a license to display the preprint in perpetuity. It is made available under aCC-BY 4.0 International license.

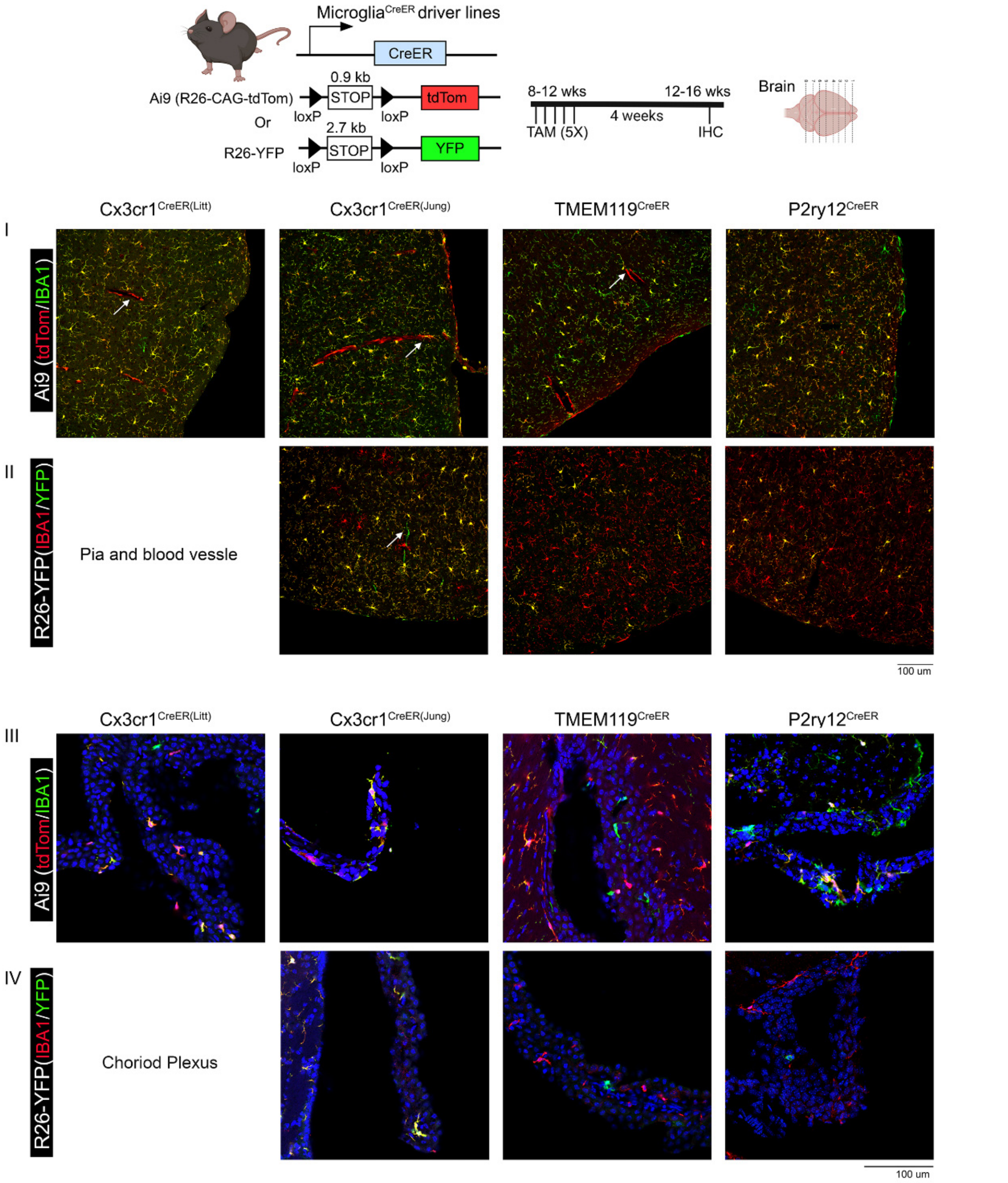

Supplementary Figure 1. Border Associated macrophage (BAMs) labeling in the four different creER driver lines using either the Ai9 (tdTomato) or R26-YFP reporter mouse lines. The experimental timeline is shown on top of the panel. Representative images from each cre driver and Ai 9 (I) or R26-YFP (II) reporter line in pia and blood vessel associated macrophages and choroid plexus associated macrophages (III Ai tdTomato) or (IV R26-YFP) reporter. Scale bar = 100µm.

bioRxiv preprint doi: https://doi.org/10.1101/2023.04.17.536878; this version posted April 17, 2023. The copyright holder for this preprint (which was not certified by peer review) is the author/funder, who has granted bioRxiv a license to display the preprint in perpetuity. It is made available under aCC-BY 4.0 International license.

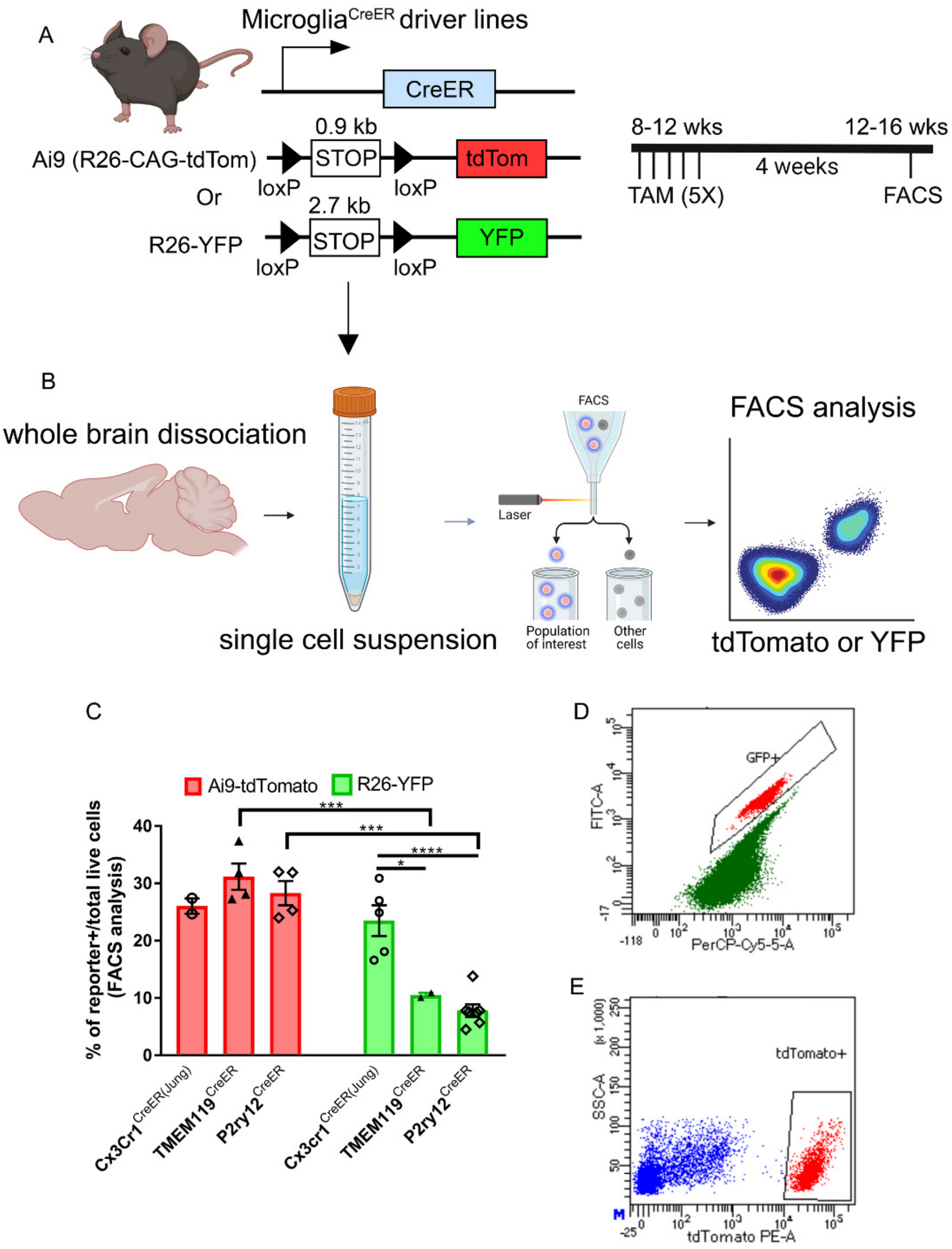

Supplementary Figure 2. FACS analysis of reporter-positive cells in total brain single cell resuspension confirms the reporter recombination efficiency differences among the three investigated CreER lines. tdTomato+ cell percentage from all three different lines show similar high-efficiency recombination and the YFP+ cells show higher recombination efficiency in the CX3CR1CreERJung-R26-YFP mice but significantly lower recombination efficiency in the P2RY12^{CreER}-R26-YFP mice and TMEM119^{CreER}-R26-YFP line. Each data point represents data from one animal. **p < 0.01 and ***p < 0.001, for Two way ANOVA analysis, Tukey post-hoc pairwise analysis. Data were combined from 2-3 independent cohorts of mice for each line.

bioRxiv preprint doi: https://doi.org/10.1101/2023.04.17.536878; this version posted April 17, 2023. The copyright holder for this preprint (which was not certified by peer review) is the author/funder, who has granted bioRxiv a license to display the preprint in perpetuity. It is made available under aCC-BY 4.0 International license.

Supplementary Table 1. Double reporter labeling of P2ry12CreER(+/WT) after TAM

Cell Condition	Mouse 1	Mouse 2	Mouse 3	Average
% of total tdTomato+	77.77778	88.09524	81.69014	82.52105
% of total YFP+	45.29915	35.71429	43.66197	41.55847
% of YFP+:tdTomato-	22.22222	11.90476	18.30986	17.47895
% of YFP-:tdTomato+	54.70085	64.28571	56.33803	58.44153
% of YFP+:tdTomato+	23.07692	23.80952	25.35211	24.07952

*All percentages are calculated by dividing the number of each category of cells with the total reporter positive cells from each animal.

Supplementary Table 1. Quantification of the FACS analysis of the reporter+ microglia in whole brain single cell suspension prepared from the P2RY12CreER-Ai9-R26-YFP mice after TAM treatment.
